# Human Immunodeficiency Virus Infection and Diverse Physical Health Outcomes: An Umbrella Review of Meta-analyses of Observational Studies

**DOI:** 10.1093/cid/ciz539

**Published:** 2019-08-11

**Authors:** Igor Grabovac, Nicola Veronese, Sinisa Stefanac, Sandra Haider, Sarah E Jackson, Ai Koyanagi, Michael Meilinger, Brendon Stubbs, Joseph Firth, Pinar Soysal, Francesco Di Gennaro, Jacopo Demurtas, Daragh T McDermott, Adam D Abbs, Lin Yang, Lee Smith

**Affiliations:** 1 Department of Social and Preventive Medicine, Center for Public Health, Medical University of Vienna, Austria; 2 Neuroscience Institute, Aging Branch, National Research Council, Padua, Italy; 3 Institute of Outcomes Research, Center for Medical Statistics, Informatics and Intelligent Systems, Medical University of Vienna, Austria; 4 Department of Behavioural Science and Health, University College London, United Kingdom; 5 Parc Sanitari Sant Joan de Déu, Universitat de Barcelona, Fundació Sant Joan de Déu, Centro de Investigación Biomédica en Red de Salud Mental, Spain; 6 2nd Department of Respiratory and Critical Care, Otto Wagner Hospital, Vienna, Austria; 7 Physiotherapy Department, South London and Maudsley National Health Service Foundation Trust, London, United Kingdom; 8 National Institute of Complementary Medicine Health Research Institute, Western Sydney University, Westmead, Australia; 9 Department of Geriatric Medicine, Bezmialem Vakif University, Faculty of Medicine, Istanbul, Turkey; 10 Clinic of Infectious Diseases University of Bari, Italy; 11 Primary Care Department, Azienda Usl Toscana Sud Est, Grosseto, Italy; 12 School of Psychology and Sport Science, Anglia Ruskin University, Cambridge, United Kingdom; 13 Pennine Acute Hospitals, NHS Trust, United Kingdom; 14 Department of Cancer Epidemiology and Prevention Research, Alberta Health Services, Holy Cross Centre, Canada; 15 Preventive Oncology & Community Health Sciences, Cumming School of Medicine, University of Calgary, Canada; 16 The Cambridge Centre for Sport and Exercise Sciences, Anglia Ruskin University, United Kingdom

**Keywords:** HIV, human immunodeficiency virus, health outcomes, comorbid, umbrella review

## Abstract

**Background:**

Our aim was to assess both the credibility and strength of evidence arising from systematic reviews with meta-analyses of observational studies and physical health outcomes associated with human immunodeficiency virus (HIV) but not acquired immunodeficiency syndrome.

**Methods:**

We performed an umbrella review of observational studies. Evidence was graded as convincing, highly suggestive, suggestive, weak, or nonsignificant.

**Results:**

From 3413 studies returned, 20 were included, covering 55 health outcomes. Median number of participants was 18 743 (range 403–225 000 000). Overall, 45 (81.8%) of the 55 unique outcomes reported nominally significant summary results (*P* < .05). Only 5 outcomes (9.0%; higher likelihood of presence of breathlessness, higher chronic obstructive pulmonary disease [COPD] prevalence, maternal sepsis, higher risk of anemia, and higher risk of all fractures among people living with HIV [PLWHIV]) showed suggestive evidence, with *P* values < 10^–3^; only 3 (5.5%; higher prevalence of cough in cross-sectional studies, higher incidence of pregnancy-related mortality, and higher incidence of ischemic heart disease among PLWHIV in cohort studies) outcomes showed stronger evidence using a stringent *P* value (<10^–6^). None of the unique outcomes presented convincing evidence (Class I), yet 3 outcomes presented highly suggestive evidence, 5 outcomes presented suggestive evidence, and 37 outcomes presented weak evidence.

**Conclusions:**

Results show highly suggestive and suggestive evidence for HIV and the presence of a cough, COPD, ischemic heart disease, pregnancy-related mortality, maternal sepsis, and bone fractures. Public health policies should reflect and accommodate these changes, especially in light of the increases in the life expectancy and the incidence of comorbidities in this population.

## Teaser

This umbrella review captures the complexities and variety of physical outcomes associated with human immunodeficiency virus. It reports on 55 unique outcomes: 3 (higher prevalence of cough and higher incidences of pregnancy-related mortality and ischemic heart disease) had strong evidence.

The incidence rates of human immunodeficiency virus (HIV) infection have shown a slow but steady decline over the past 2 decades. Despite this decline, 1.8 million people globally are newly infected with HIV every year, which maintains HIV’s status as a top global health issue [1].

Globally, the most common cause of death in people living with HIV (PLWHIV) is tuberculosis [2]. However, in developed countries where tuberculosis is rare and there is high access to antiretroviral treatment (ART), diseases not related to acquired immunodeficiency syndrome (AIDS; including but not limited to diabetes mellitus, kidney disease, liver disease, hypertension, cardiovascular disease, and non–AIDS related malignancies) are a leading cause of death among PLWHIV who receive treatment [3]. Correspondingly, the importance of non–AIDS related comorbidities, complications, and mortality has increased over the last decade [4].

Reasons for the rise in non–AIDS related morbidity is a question of some debate. In general, the availability of ART led to a substantial rise in the life expectancy of PLWHIV, which is now almost comparable to that of the general, seronegative population and may be accompanied by more non–AIDS related morbidity [5]. Studies have, however, reported greater prevalences of age-associated diseases in PLWHIV. This may be explained by the persistent immunodeficiency, immune activation, and chronic infection–associated inflammation connected with infection [6]. Additionally, ART itself may act directly, with reported adverse effects including hyperlipidaemia, insulin resistance, and the proinflammatory effect of some nucleoside analogues [7, 8]. Lastly, PLWHIV in developed countries, as a population, often exhibit greater risk factors associated with incidences of non–AIDS related illnesses, such as smoking, drug use, and alcohol use [5, 7, 9, 10].

Given the incidence, morbidity, and mortality rates associated with HIV, numerous systematic reviews and meta-analyses have attempted to quantify this disparate literature. To date, most systematic reviews have focused on a single disease end point and there has not been a systematic evaluation of the relationships between HIV and diverse physical health outcomes. Moreover, the strength and reliability of the literature is unclear.

In order to address the breadth of the literature of complex health behaviors and outcomes, an increasing emphasis has been placed on “umbrella reviews” [11] (ie, the syntheses of existing systematic reviews with meta-analyses, to capture the breadth of outcomes associated with a given exposure). In this study, we undertook an umbrella review of existing systematic reviews with meta-analyses of HIV and all physical health outcomes in order to systematically assess the quality and strength of the evidence across all health outcomes and to identify those studies with the strongest evidence.

## MATERIALS AND METHODS

The protocol for the present umbrella review was preregistered with PROSPERO (registration number CRD42018107336).

An umbrella review was carried out following standardized procedures [11, 12]. Electronic databases were systematically searched via MEDLINE/PubMed, PsycINFO, and Embase from inception to 25 August 2018. The following search terms were used in MEDLINE/PubMed: “(meta-analysis or meta-anal* or systematic review) and (HIV or LAV or HTLV III or HTLV-III or AIDS or human immunodeficiency virus or human T-lymphotropic virus III or acquired immunodeficiency).” In addition, we hand-searched the reference lists of eligible articles and other narrative overviews of systematic reviews/meta-analyses.

The primary screening was carried out by 2 authors (I. G. and L. Y.) and any disagreements were resolved via screening of the disputed title/abstract by a third author (N. V.). Full texts were sourced for all potential eligible articles and were screened by 2 investigators (I. G. and L. Y.), who determined the final references to be included.

We included systematic reviews with meta-analyses of observational studies (cross-sectional, case control, or retrospective and prospective cohort studies) that investigated the relationship between HIV and any physical health outcomes. Specific inclusion criteria included: (1) systematic reviews with meta-analyses reporting data on HIV (diagnosed through self-reports or laboratory confirmation); and (2) meta-analyses of cross-sectional or cohort studies that investigated the association of HIV with any health outcome (eg, cardiovascular disease, cancer, obesity/overweight, diabetes, or metabolic diseases). Studies had to report these outcomes as odds ratios, relative risks (RRs), hazard ratios, or continuous data (standardized mean difference, weighted mean difference, or mean difference [MD]). Studies could have been published in any language. Studies were excluded if they were related to immunosuppression or related to correlates of HIV infection.

### Data Extraction

The following information was extracted from each article by 2 independent investigators: (1) first author name; (2) year of publication; (3) journal; (4) number of included studies and total number of people included in the review; (5) inclusion criteria for the studied population; (6) effect size(s) used in the review; (7) subgrouping used in the meta-analysis (with or without ART); (8) study design (case-control, retrospective, prospective); and (9) number of cases (ie, those having the outcome of interest: eg, cancer) and controls (ie, those not having the outcome of interest: eg, no cancer) for each study. We then extracted the study-specific, estimated RR for the health outcome (risk ratio, odds ratio, hazard ratio, standardized mean difference, weighted mean difference, MD), along with the 95% confidence interval (CI). If 2 meta-analyses were available for the same association, we included the largest in terms of the number of studies.

### Risk of Bias (Quality) Assessment

Independently, 2 authors rated the methodological quality of the included meta-analyses using the AMSTAR 2 tool [13] (see Supplementary Table 1 for a full list of questions asked).

### Data Analysis and Credibility Assessment

For each meta-analysis, we estimated the summary effect size and 95% CI through random-effects models [14]. In cases where the summary effect size of a meta-analysis was reported as MD, we transformed the metric to RR using an established formula [15]. We also estimated the prediction interval and its 95% CI, which further accounted for between-study effects and estimated the certainty of the association if a new study addressed that same association [16–18]. For the largest data set of each meta-analysis, we calculated the standard error (SE) of the effect size. If the SE was less than 0.10, then the 95% CI would be lower than 0.20 (which is less than the magnitude of a small effect size). We also considered whether the largest study was significant (ie, *P* value < .05). The between-study association was estimated with the I^2^ metric; values > 50% were indicative of high heterogeneity, while values above 75% suggest very high heterogeneity [19, 20].

In addition, we calculated the evidence of small-study effects (ie, whether small studies would have inflated effect sizes compared to larger studies). To this end, we used the regression asymmetry test developed by Egger and coworkers [21]. A *P* value < .10, with more conservative effects in larger studies than in random-effects meta-analyses, was considered as indicative of small-study effects [22].

Finally, we applied the excess significance test [23], which evaluates whether the number of studies with nominally significant results (ie, with *P* < .05) among those included in a meta-analysis is too large, based on the power that these data sets have to detect effects at α = .05. For this analysis, the power estimate for each data set was calculated. The sum of the power estimates of each study provided the expected number of data sets with nominal statistical significance. As described elsewhere, the number of expected “positive” (ie, statistically significant data sets) studies can be compared with the observed number of statistically significant studies through a χ ^2^-based test [23]. The larger the difference between the observed and expected number of significant studies, the higher the degree of excess of a significance bias. The true effect size of a meta-analysis is unknown. We considered the effect size of the largest study for each meta-analysis (ie, with the lower SE) and, based on this, we estimated the effect sizes of each constituent study with an algorithm using a noncentral *t* distribution. The excess significance for a single meta-analysis was considered whenever the *P* value was < .10.

We used credibility assessment criteria (based on established tools for observational evidence, as summarized previously [24, 25]) and classified the evidence of significant outcomes (*P* value in random effect model <.05) in 4 classes (Class I, convincing; Class II, highly suggestive; Class III, suggestive; Class IV, weak). Full details are given in Table 1.

**Table 1.** Credibility Assessment Criteria for Meta-analyses of Observational Studies

Evidence Classification	Criteria
Convincing (Class I)	Associations with *P* < .000001; >1000 cases (or >20 000 participants for continuous outcomes) having the event of interest; the largest component study reporting a nominal, statistically significant result (*P* < .05); a 95% prediction interval that excluded the null; no large heterogeneity (I^2^ < 50%); no evidence of small-study effect (*P* > .10); no excess significance bias (*P* > .10).
Highly suggestive (Class II)	Associations with *P* < .000001; >1000 cases (or >20 000 participants for continuous outcomes) having the event of interest; the largest component study reporting a statistically significant result (*P* < .05).
Suggestive (Class III)	Associations with *P* < .001; >1000 cases (or >20 000 participants for continuous outcomes) having the event of interest.
Weak (Class IV)	Remaining statistically significant associations with *P* < .05.

## RESULTS

**Figure 1.**
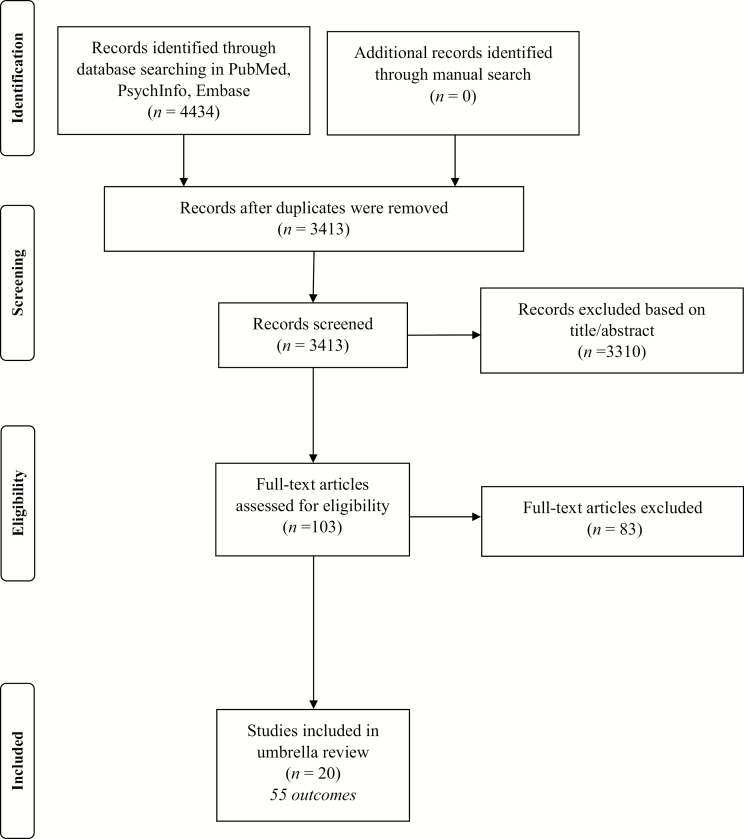
Preferred Reporting Items for Systematic Reviews and Meta-Analyses flowchart.

As shown in Figure 1, the review of the literature identified 3413 unique papers across 3 major databases. After applying the inclusion/exclusion criteria, 103 papers were identified and, of them, 20 were eligible. A total of 55 unique health outcomes were assessed across the 20 meta-analyses included in our umbrella review.

### Meta-analyses of Observational Studies

As reported in Supplementary Table 2, the median number of studies for each outcome in meta-analyses was 8 (range 2–42), the median number of participants was 18 743 (range 403–225 000 000), and the median number of cases was 847 (range 7–627 199). The diverse comorbidity outcomes included metabolic disorders, respiratory conditions, mortality, cardiovascular conditions, cancer, and maternal and newborn outcomes.

Overall, for 45 (81.8%) of the 55 unique outcomes, there were nominally significant summary results (*P* < .05); among these, 5 outcomes (9.0%) showed suggestive evidence, with *P* values < 10^–3^, and 3 (5.5%) outcomes showed stronger evidence using a stringent *P* value (<10^–6^).

Heterogeneity among studies was generally high: 39/55 outcomes (70.9%) had an estimated I^2^ consistent with large heterogeneity (>50%), with 26 showing very large heterogeneity (>75%). There were 5 outcomes that presented 95% prediction intervals, excluding the null value. The evidence for excess statistical significance (ie, whether smaller studies tend to give substantially larger estimates of effect size, compared with larger studies) was present in 17 outcomes (30.9%), and small-study effects were also seen in 16 of the outcomes (29.1%). Studies with the largest sample size for each outcome maintained their statistical significance in 38/55 outcomes (69.1%). Further, for 15/55 outcomes (27.3%), the largest study had a more conservative effect, compared to the random-effects model.

Based on the above criteria, no outcome presented convincing evidence (Class I). There were 3 outcomes that presented highly suggestive evidence (Class II: higher prevalence of cough in cross-sectional studies, higher incidence of pregnancy-related mortality, and higher incidence of ischemic heart disease among PLWHIV in cohort studies), 5 outcomes that presented suggestive evidence (Class III: higher likelihood of presence of breathlessness, higher chronic obstructive pulmonary disease [COPD] prevalence, maternal sepsis, higher risk of anemia, and higher risk of all fractures among PLWHIV; Tables 1 and 2), and 37 outcomes that presented weak evidence. The 3 outcomes in Class II scored low or critically low according to the AMSTAR 2 evaluation (Supplementary Table 1).

**Table 2.** Summarized Physical Health Outcomes and Evidence Class Reported in Included Meta-analyses of Observational Studies

	Level of Evidence				
Outcome	I	II	III	IV	NS
CVD	None	Cohort studies: risk of ischemic heart disease	None	Cohort studies: risk of AMI, CAD, any stroke, ischemic stroke	Case-control studies: risk of coronary stenosis; risk of calcified plaques; risk of any plaque
				Case-control studies: risk of noncalcified plaques	…
				Case-control/cross-sectional studies: prevalence of IMT, flow-mediated vasodilatation, pulse wave velocity	…
Gynecological outcomes	None	Cohort studies: risk of pregnancy-related mortality	None	Cohort studies: risk of stillbirth, perinatal mortality, infant mortality, pregnancy-induced hypertension, caesarian sepsis, caesarian wound infection, caesarian endometritis, low birthweight, preterm delivery	Cohort studies: risk of neonatal mortality, pregnancy-related hypertension, pre-eclampsia, eclampsia, abnormal presentation, caesarian section
				Cohort/case-control studies: risk of uterine rupture, endometritis	…
Metabolic and blood pressure outcomes	None	None	None	Cohort studies: mean hemoglobin levels	…
				Cross-sectional/case-control studies: mean BMI, TGs, HDL, SBP, DBP levels	Cross-sectional/case-control studies: mean LDL, glucose, HbA1c levels
Other outcomes	None	Cross-sectional studies: prevalence of cough	Cohort studies: risk of fractures, sepsis, death	Cohort studies: risk of intracranial hemorrhage, renal disease, melanoma (pre- and post-HAART time period)	…
			…	Case-control studies: risk of intracerebral hemorrhage	…
			Cross-sectional studies: prevalence of breathlessness, anemia, CPPD	Cross-sectional studies: prevalence of ED.	Cross-sectional/case-control studies: prevalence of dental caries

Abbreviations: AMI, acute myocardial infarction; BMI, body mass index; CAD, coronary artery disease; CPPD, calcium pyrophosphate dihydrate crystal deposition disease; CVD, cardiovascular disease; DBP, diastolic blood pressure; ED, erectile dysfunction; HAART, highly active antiretroviral therapy; HbA1c, glycated hemoglobin; HDL, high-density lipoprotein; IMT, intima media thickness; LDL, low-density lipoprotein; NS, not significant; SBP, systolic blood pressure; TGs, triglycerides.

The majority of meta-analyses scored critically low (*n* = 15) on AMSTAR 2, and 4 scored low (Supplementary Table 1).

## DISCUSSION

In this study, which included 20 meta-analyses and 55 different outcomes that are associated with HIV infection, we found highly suggestive evidence that HIV infection is associated with a higher presence of coughing in cross-sectional studies and higher risks of pregnancy-related mortality and ischemic heart disease in cohort studies. Suggestive evidence was found for a higher likelihood of the presence of breathlessness, a higher COPD prevalence, maternal sepsis, a higher risk of anemia, and a higher risk of all fractures among PLWHIV. These conclusions are based on the evaluation of epidemiological evidence credibility, which is a common approach used in a variety of research. Such critical appraisals of literature are necessary, as the nominal significance level of *P* < .05 is widely used to claim new associations in literature. However, emerging evidence shows that results based on this criterion constitute weak evidence, as also confirmed by our umbrella review, where 45 outcomes were statistically significant (*P* < .05) but no convincing evidence was evident: highly suggestive evidence was observed for only 3 outcomes.

We found highly suggestive evidence that HIV infection is associated with the presence of coughing and suggestive evidence for an association with the presence of breathlessness and the prevalence of COPD. These results indicate that, even with the high availability of ART, PLWHIV experience disproportionally more chronic respiratory illness in comparison to seronegative populations [26]. This may be linked to the exponential rise in life expectancy for PLWHIV [27, 28], bringing about a population of aging PLWHIV that have higher prevalences of comorbidities and respiratory illnesses. Additionally, PLWHIV in resource-rich settings have higher prevalences of smoking and illicit drug use than seronegative people [9, 10, 29, 30], which are linked to higher respiratory and cardiovascular disease incidences [31].

Our analysis also showed highly suggestive evidence of the association between HIV infection and ischemic heart disease. Reasons for this include the aforementioned high prevalences of smoking and illicit drug use within this population, but may also be related to underlying, chronic inflammation and immune activation, combined with coagulation abnormalities and atherosclerosis [32]. Overall, following the advent of ART, the mortality of PLWHIV attributable to cardiovascular disease is considerable [33]. Studies indicate that PLWHIV have a higher risk of cardiovascular disease, in comparison to seronegative people, with some earlier studies indicating an even higher risk among PLWHIV receiving protease inhibitors, in comparison to therapy-naive PLWHIV [34, 35]. Reasons for this may be linked to “inflamm-aging”: a term that describes a chronic, low-grade inflammation characteristic of biological aging that shares similarities with the persistent immune activation observed in PLWHIV [36–39]. The resulting immunosenesence coupled with “inflamm-aging” may predispose PLWHIV to comorbid conditions such as cardiovascular disease, metabolic and neurocognitive disorders, or cancer [37, 38, 40]. Reports on the specific pathophysiological processes leading to lung symptoms are rare, but may be explained by the reduced ability of alveolar macrophages to maintain homeostasis [41].

We report on highly suggestive and suggestive levels of evidence concerning the association of HIV with pregnancy-related mortality and sepsis, respectively. The pathological interactions between HIV infection and pregnancy-related outcomes are poorly understood [42, 43]; however, given the high number of women living with HIV, with the highest proportion in resource-poor settings where there is a substantial risk of maternal mortality, there is an intersecting problem that poses a major public health issue [1]. Systematic reviews have found no evidence to support the idea that pregnancy somehow accelerates HIV’s progression. Our analysis did show highly suggestive evidence to support the association of maternal HIV infection and pregnancy-related mortality. This may be explained by the generally poor health of women living with HIV, but may also be due to HIV-related thrombocytopenia, leading to excessive bleeding [44, 45]. Moreover, high levels of stigma and other social issues faced by women living with HIV might prevent or inhibit their access to healthcare services [46, 47]. Lower immune activation and suppression may be the underlying reason for the high susceptibility to infections and sepsis in women living with HIV, with the latter showing a suggestive level of evidence in our analysis [48, 49].

The final 2 outcomes showing suggestive levels of evidence in our analysis were the risk of anemia in children and the risk of bone fractures. For both outcomes, the mechanisms remain unclear. While anemia has been reported to be among the most common hematological complications to follow HIV infection, bone fractures have not been as commonly reported. The literature suggests effects of HIV on erythrocyte production in cases of anemia [50], while the effects on bone loss and fractures seem to be less clear, with most studies reporting diverse variables that may have synergistic effects, such as tobacco smoking, drug use, low body weights, low CD4+ cell counts, and hepatitis C coinfections [51].

Umbrella reviews provide top-tier evidence and important insights, but there are a number of limitations that need to be considered. The meta-analyses contained studies that differed in their designs, populations, and other characteristics. However, we applied an I^2^ <50% as 1 of the criteria for Class I evidence (convincing), in order to assign the best evidence grade only to robust associations. Second, many studies in our umbrella review determined individuals’ HIV statuses using self-reported information, but the agreement between self-reported and bio-humoral diagnoses is often poor [52]. Finally, meta-analyses have inherent limitations [53]: their findings depend on which estimates are selected from each primary study and how they are applied in the meta-analysis.

## CONCLUSION

Our results show highly suggestive and suggestive evidence for links between HIV and the presence of coughs, prevalence of COPD, incidence of ischemic heart disease, incidence of pregnancy-related mortality, incidence of maternal sepsis, and risk of bone fractures. Public health policies should reflect and accommodate these changes, especially in light of the increases in both life expectancy and incidences of comorbidities in this population. The elevated risks of the reported outcomes requires more research to improve both the prevention and early detection of these comorbidities in PLWHIV.

## Supplementary Data

Supplementary materials are available at *Clinical Infectious Diseases* online. Consisting of data provided by the authors to benefit the reader, the posted materials are not copyedited and are the sole responsibility of the authors, so questions or comments should be addressed to the corresponding author.

ciz539_suppl_Supplementary_Table-1

ciz539_suppl_Supplementary_Table-2

## Notes

***Author contributions.*** L. S. conceived the idea for this study. I. G., L. Y., L. S., and N. V. designed the protocol, carried out searches and screening, and analyzed the data. I. G., L. Y., L. S., S. S., and S. H. extracted the data. I. G., L. S., L. Y., and N. V. drafted the manuscript. All authors provided critical revisions and approved the final version of the manuscript.

***Potential conflicts of interest.*** The authors: No reported conflicts of interest. All authors have submitted the ICMJE Form for Disclosure of Potential Conflicts of Interest. Conflicts that the editors consider relevant to the content of the manuscript have been disclosed.
