# Supplementary material for: Human Immunodeficiency Virus Infection and Diverse Physical Health Outcomes: An Umbrella Review of Meta-analyses of Observational Studies
Source: Clin Infect Dis. 2019 Aug 11;70(9):1809–15. doi: 10.1093/cid/ciz539 (PMC7156772; doi:10.1093/cid/ciz539)
Supplement: ciz539_suppl_Supplementary_Table-2 [file ciz539_suppl_supplementary_table-2.docx]

**Supplementary table 2. Health outcomes and evidence class reported in included meta-analyses of observational studies.**

| **Author,[reference]** | **year** | **Outcome** | **Study design** | **N of  studies** | **Cases** | **Sample  size** | **ES** | **Mean ES  (95%CI)** | **P^a^** | **95% prediction interval^b^** | **I^2^** | **p-value Egger** | **Observed/ expected significant studies** | **Excess significance test** | **Level of evidence** |
| --- | --- | --- | --- | --- | --- | --- | --- | --- | --- | --- | --- | --- | --- | --- | --- |
| Brown | 2016 | Presence of cough | Cross-sectional | 19 | 3547 | 30939 | OR | 2.138 (1.614-2.832) | 1.17E-07 | 0.64, 7.14 | 88.60% | 0 | 14.28/3 | <0.0001 | II |
| Calvert | 2013 | pregnancy related mortality | Cohort | 23 | 3478 | 8982338 | RR | 7.774(5.399-11.194) | 2.88E-28 | 2.45, 24.64 | 53.80% | 0.396 | 13.75/0 | <0.0001 | II |
| Islam | 2012 | ischaemic heart disease | Cohort | 3 | 27503 | 1426249 | RR | 1.606(1.426-1.808) | 5.83E-15 | 0.61, 4.26 | 18.20% | 0.905 | 0.29/0 | 1 | II |
| Brown | 2016 | Presence of breathlessness | Cross-sectional | 12 | 1704 | 12242 | OR | 1.796(1.289-2.501) | 5.32E-04 | 0.61, 5.3 | 78.10% | 0.015 | 6.83/0 | <0.0001 | III |
| Calis | 2008 | Risk of anemia | Cross-sectional | 6 | 1636 | 2977 | OR | 4.507(2.447-8.302) | 1.35E-06 | 0.68, 29.95 | 73.30% | 0.718 | 4.84/1 | <0.0001 | III |
| Shiau | 2013 | All fractures | Cohort | 4 | 10355 | 225,000,000 | RR | 1.579(1.246-2.001) | 0.00015426 | 0.63, 3.95 | 57.30% | 0.998 | 4/3 | 0.05 | III |
| Bigna et al | 2018 | COPD prevalence | Cross-sectional | 11 | 16196 | 327801 | OR | 1.16(1.066-1.263) | 0.00059631 | 0.94, 1.43 | 58.40% | 0.892 | 6.18/3 | 0.07 | III |
| Calvert | 2013 | Sepsis | Cohort | 4 | 79474 | 8801088 | OR | 3.429(2.047-5.745) | 2.87E-06 | 0.66, 17.7 | 8.60% | 0.633 | 2/0 | 0.04 | III |
| Gutierrez | 2017 | risk of death | Cohort | 6 | NP | 3522019 | HR | 3.641(2.123-6.245) | 2.67E-06 | 0.54, 24.36 | 97.60% | 0.941 | NP | NP | IV |
| Gutierrez | 2017 | risk of myocardial infarction | Cohort | 7 | NP | 1440789 | HR | 1.629(1.412-1.879) | 2.30E-11 | 1.09, 2.44 | 63.80% | 0.596 | NP | NP | IV |
| Gutierrez | 2017 | risk of coronary artery disease | Cohort | 9 | NP | 4885940 | HR | 1.517(1.207, 1.906) | 0.00035329 | 0.7, 3.29 | 93.30% | 0.017 | NP | NP | IV |
| Gutierrez | 2017 | any stroke | Cohort | 3 | NP | 53635 | HR | 1.819(1.528, 2.165) | 1.71E-11 | 0.59, 5.64 | 0.00% | 0.646 | NP | NP | IV |
| Gutierrez | 2017 | Ischemic stroke | Cohort | 4 | NP | 522867 | HR | 1.278 (1.138, 1.434) | 3.1594E-05 | 0.88, 1.85 | 28.50% | 0.201 | NP | NP | IV |
| Gutierrez | 2017 | Intracranial hemorrhage | Cohort | 3 | NP | 1132506 | HR | 2.22 (1.593, 1.434) | 2.49E-06 | 0.2, 24.74 | 7.70% | 0.332 | NP | NP | IV |
| Brocklehurst | 1998 | Stillbirth | Cohort | 8 | 889 | 29746 | OR | 2.02 (0.79, 5.1666) | 1.42E-01 | 0.1, 39.66 | 77.10% | 0.068 | 1.36/1 | 1 | IV |
| Brocklehurst | 1998 | Perinatal mortality | Cohort | 9 | 202 | 27766 | OR | 1.546 (0.999, 2.395) | 3.01E-02 | 0.59, 4.05 | 27.50% | 0.022 | 2.8/0 | 0.07 | IV |
| Brocklehurst | 1998 | Infant mortality | Cohort | 9 | 853 | 6836 | OR | 3.933 (2.856, 2.053) | 4.81E-17 | 1.81, 8.54 | 41.80% | 0.354 | 7.7/1 | <0.0001 | IV |
| Calvert | 2013 | Pregnancy induced hypertension | Cohort | 11 | 627199 | 8836983 | OR | 1.455 (1.031, 2.053) | 3.30E-02 | 0.52, 4.06 | 79.30% | 0.726 | 5.22/2 | 0.06 | IV |
| Calvert | 2013 | Uterine rupture | Cohort/case-control | 2 | 71 | 23386 | OR | 3.135 (1.512, 6.5) | 2.13E-03 | NP | 0.00% | NP | 1/0 | <0,0001 | IV |
| Calvert | 2013 | Endometritis | Cohort/case-control | 8 | 81 | 2268 | OR | 2.519 (1.509, 4.205) | 0.00040821 | 0.93, 6.85 | 18.80% | 0.748 | 3.72/0 | <0.0001 | IV |
| Calvert | 2013 | Caesarian- sepsis | Cohort | 4 | 7 | 743 | OR | 5.817 (2.42, 13.986) | 8.3374E-05 | 0.85, 39.9 | 0.00% | 0.484 | 1.02/0 | 0.58 | IV |
| Calvert | 2013 | Caesarian - wound infection | Cohort | 10 | 906 | 57392 | OR | 1.746 (1.194, 2.551) | 0.00401302 | 0.74, 4.14 | 30.00% | 0.087 | 4.57/0 | <0.0001 | IV |
| Calvert | 2013 | Caesarian- endometritis | Cohort | 12 | 3478 | 58229 | OR | 1.866 (1.284, 2.713) | 0.00107965 | 0.7, 4.95 | 47.00% | 0.414 | 5.39/0 | <0.0001 | IV |
| D'Ascenzo | 2015 | Risk of non-calcified plaques more than 0 | Case-control | 4 | 600 | 1172 | OR | 3.262 (1.302, 8.173) | 0.01162414 | 0.05, 204.15 | 83.60% | 0.091 | 3.15/0 | <0.0001 | IV |
| Xiao | 2015 | Low birthweight | Cohort | 43 | NR | 21465 | OR | 1.98 (1.757, 2.23) | 2.82E-29 | 1.08, 3.64 | 71.80% | 0 | NP | NP | IV |
| Xiao | 2015 | Preterm delivery | Cohort | 40 | NR | 27281 | OR | 1.623 (1.488, 1.77) | 1.11E-27 | 1.15, 2.29 | 51.90% | 0.156 | NP | NP | IV |
| Behrouz et al | 2016 | Risk of intracerebral hemorrhage | Case-control | 6 | 841 | 177719 | RR | 3.896 (2.037, 7.449) | 3.9262E-05 | 0.47, 32.32 | 85.20% | 0.181 | 5.55/1 | <0.0001 | IV |
| Islam_a | 2012 | renal disease | Cohort | 3 | NR | 3227 | RR | 3.867 (2.183, 6.848) | 3.53E-06 | 0.01, 1053.69 | 43.80% | 0.489 | NP | NP | IV |
| Olsen | 2014 | Melanoma, in the post-HAART time period | Cohort | 6 | 284 | 702897 | RR | 1.501 (1.123, 2.006) | 0.00611078 | 0.68, 3.31 | 54.50% | 0.292 | 1.93/1 | 0.67 | IV |
| Olsen | 2014 | Melanoma, in the pre-HAART time period | Cohort | 3 | 235 | 1054700 | RR | 1.282 (1.104, 1.489) | 0.0011601 | 0.5, 3.22 | 0.00% | 0.295 | NP | 0.5 | IV |
| Luo | 2017 | Prevalence of ED | Cross-sectional | 5 | 690 | 4252 | RR | 2.438 (1.588, 3.745) | 4.659E-05 | 0.56, 10.63 | 87.30% | 0.779 | 2.39/2 | 0.49 | IV |
| Dillon | 2013 | BMI | Cross-sectional/case-control | 36 | NR | 24725 | SMD | -0.319 (-0.452, -0.186) | 2.66E-06 | -1.06, 0.44 | 93.40% | 0.684 | NP | NP | IV |
| Dillon | 2013 | TGs | Cross-sectional/case-control | 15 | NR | 13198 | SMD | 0.261 (0.085, 0.437) | 0.00372672 | -0.16, 0.98 | 91.30% | 0.128 | NP | NP | IV |
| Dillon | 2013 | HDL | Cross-sectional/case-control | 14 | NR | 12710 | SMD | -0.586 (-0.86, -0.312) | 2.7353E-05 | -1.72, 0.55 | 96.20% | 0.072 | NP | NP | IV |
| Dillon | 2013 | SBP | Cross-sectional/case-control | 15 | NR | 11909 | SMD | -0.4 (-0.554, -0.247) | 3.21E-07 | -0.97, 0.17 | 84.10% | 0.279 | NP | NP | IV |
| Dillon | 2013 | DBP | Cross-sectional/case-control | 15 | NR | 11909 | SMD | -0.339 (-0.509, -0.168) | 9.7975E-05 | -0.99, 0.31 | 87.80% | 0.044 | NP | NP | IV |
| Calis | 2008 | Mean hemoglobin levels | Cohort | 5 | NR | 2228 | SMD | -0.783 (-1.097, -0.47) | 9.44E-07 | -1.89, 0.33 | 81.40% | 0.489 | NP | NP | IV |
| Sun | 2015 | Intima-media thickness | Cross-sectional/case-control | 28 | 5360 | 16021 | WMD | 0.049 (0.03, 0.067) | 2.23E-07 | -0.04, 0.14 | 96.90% | 0.083 | NP | 1 | IV |
| Sun | 2015 | Pulse wave velocity | Cross-sectional/case-control | 13 | 1547 | 2838 | WMD | 0.537 (0.283, 0.792) | 3.3837E-05 | -0.38, 1.45 | 83.90% | 0.188 | NP | 1 | IV |
| Sun | 2015 | Flow mediated vasodilation | Cross-sectional/case-control | 12 | 553 | 1099 | WMD | -1.858 (-2.623, -1.092) | 2.01E-06 | -4.34, 0.63 | 88.90% | 0.622 | NP | 1 | IV |
| Brocklehurst | 1998 | Neonatal mortality | Cohort | 3 | 51 | 1517 | OR | 1.153 (0.596, 2.228) | 2.63E-01 | 0, 340.39 | 25.10% | 0.003 | 0.56/0 | 1 | NS |
| Browne | 2015 | Risk of developing pregnancy related hypertension | Cohort | 14 | 2790 | 24335 | RR | 1.263 (0.872, 1.828) | 2.17E-01 | 0.36, 4.47 | 78.60% | 0.201 | 7.32/5 | 0.29 | NS |
| Browne | 2015 | Risk of developing pre-eclampsia | Cohort | 17 | NR | NR | RR | 1.013 (0.868, 1.182) | 8.73E-01 | 0.66, 1.55 | 63.90% | 0.252 | 6.16/4 | 0.3 | NS |
| Browne | 2015 | Risk of developing eclampsia | Cohort | 5 | 280 | 5503 | RR | 1.609 (0.139, 18.679) | 7.04E-01 | 0, 21754 | 97.00% | 0.194 | 2.72/2 | 0.67 | NS |
| Calvert | 2013 | Pre-eclampsia | Cohort | 9 | 713 | 14971 | OR | 1.036 (0.602, 1.783) | 9.00E-01 | 0.19, 5.55 | 70.70% | 0.015 | 3.32/2 | 0.5 | NS |
| Calvert | 2013 | Eclampsia | Cohort | 4 | 262 | 26550 | OR | 2.567 (0.15, 43.86) | 5.15E-01 | 0, 2045733.38 | 96.60% | 0.274 | 2.22/1 | 0.33 | NS |
| Calvert | 2013 | Abnormal presentation | Cohort | 2 | 56 | 918 | OR | 1.17 (0.676, 2.025) | 5.74E-01 | NP | 0.00% | NP | 0.21/0 | 1 | NS |
| Calvert | 2013 | Caesarian section | Cohort | 19 | 8363 | 43323 | OR | 1.202 (0.814, 1.775) | 0.354283 | 0.22, 6.56 | 88.40% | 0.002 | 7.28/4 | 0.15 | NS |
| D'Ascenzo | 2015 | Risk of coronary stenosis | Case-control | 9 | 1061 | 1672 | OR | 1.378 (0.862, 2.203) | 0.180694 | 0.31, 6.2 | 71.80% | 0.012 | 5.21/1 | 0.01 | NS |
| D'Ascenzo | 2015 | Risk of calcified plaques more than 0 | Case-control | 3 | 332 | 1045 | OR | 1.17 (0.53, 2.171) | 0.619728 | 0, 749.13 | 51.70% | 0.146 | 1.03/0 | 0.56 | NS |
| D'Ascenzo | 2015 | Risk of plaques more than 0 | Case-control | 6 | 567 | 1533 | OR | 0.874 (0.427, 1.788) | 0.711934 | 0, 9.8 | 83.10% | 0.244 | 4.44/3 | 0.19 | NS |
| Dillon | 2013 | LDL | Cross-sectional/case-control | 14 | NR | 12710 | SMD | -0.154 (-0.337, 0.029) | 0.09886 | -0.88, 0.57 | 91.20% | 0.684 | NP | NP | NS |
| Dillon | 2013 | glucose | Cross-sectional/case-control | 6 | NR | 4036 | SMD | 0.354 (-0.341, 1.05) | 0.317876 | -2.23, 2.94 | 98.50% | 0.31 | NP | NP | NS |
| Dillon | 2013 | HbA1c | Cross-sectional | 3 | NR | 6099 | SMD | -0.068 (-0.392, 0.255) | 0.678643 | -3.85, 3.71 | 82.10% | 0.803 | NP | NP | NS |
| Oliveira | 2015 | dental caries prevalence | Cross-sectional/case-control | 5 | 233 | 405 | OR | 1.58 (0.764, 3.269) | 0.217033 | 0.14, 17.57 | 64.40% | 0.011 | 2.27/1 | 0.39 | NS |
| **Summary Statistics** |  |  | **Median = 8 (range: 2-43)** | **Median = 8 (range: 2-43)** | **Median = 847 (range: 7- 627,199)** | **Median = 18,743 (range: 405 to 2.25E+08)** |  |  |  |  |  |  |  |  |  |

Abbreviations: ES: effect size; CI: confidence interval; RR: relative risk; OR: odds ratio; MD: mean difference; SMD: standardized mean difference; SMD: weighted mean difference; HR: hazard ratio; NR: not reported; NP: not possible; NS: not significant; COPD: chronic obstructive pulmonary disease; ED: erectile dysfunction; BMI: body mass index; TGs: triglycerides; HDL: high density lipoprotein; SBP: systolic blood pressure; DBP: diastolic blood pressure; LDL: low density lipoprotein; HbA1c: glycated hemoglobin

^a^ P value of summary random effects estimate.

^b^ Prediction intervals are reported only for meta-analyses including at least 3 studies.

^1^Evidence class criteria: class I (convincing): statistical significance with P<10^−6^, more than 1,000 cases (or >20,000 participants for continuous outcomes), the largest component study reported statistically significant effect (P<0.05); 95% prediction interval excluded the null; no large heterogeneity (I^2^ <50%), no evidence of small study effects (P>0.10) and excess significance bias (P>0.10); class II (highly suggestive): statistical significance with P<10^−6^, more than 1,000 cases (or >20,000 participants for continuous outcomes), the largest component study reported statistically significant effect (P<0.05); class III (suggestive): statistical significance with P<10^−3^, more than 1,000 cases (or >20,000 participants for continuous outcomes); class IV (weak): the remaining statistically significant associations with P<0.05.
